# One Health approaches adapted in low resource settings to address antimicrobial resistance

**DOI:** 10.1016/j.soh.2023.100011

**Published:** 2023-02-28

**Authors:** Ripan Biswas, Chanchal Debnath, Samiran Bandyopadhyay, Indranil Samanta

**Affiliations:** a Department of Veterinary Public Health, F/o Veterinary and Animal Sciences, West Bengal University of Animal and Fishery Sciences, 37, K. B. Sarani, Kolkata, 700037, West Bengal, India; b ICAR-Indian Veterinary Research Institute, Eastern Regional Station, Kolkata, West Bengal, India; c Department of Veterinary Microbiology, F/o Veterinary and Animal Sciences, West Bengal University of Animal and Fishery Sciences, 37, K. B. Sarani, Kolkata, 700037, West Bengal, India

**Keywords:** Antimicrobial resistance, One Health, India, Agriculture, Aquaculture, Livestock, Wildlife

## Abstract

Inter-disciplinary collaborations are now considered as key factors for integrated health system strengthening. Its application in the domain of One Health needs more milestones to achieve. Other than the human health sector, the antimicrobials are used in food animals and aquaculture for therapy, prophylaxis and growth promotion which significantly contributes to the development of antimicrobial resistance. It is the high time to develop a sustainable collaboration between the concerned sectors of One Health for a resilient health system. The domain of One Health not only mitigates the emergence of antimicrobial resistance but also helps in realizing the surveillance and epidemiology of zoonotic diseases, and the control of public health emergencies such as COVID-19. The review identified the key One Health strategies adapted by India, the exemplary low resource settings, to address antimicrobial resistance and zoonosis.

## 1 Introduction

Antimicrobial resistance (AMR) is the resistance of microorganisms to an antimicrobial to which they were once sensitive. AMR is a life threatening, serious global health problem that causes 700,000 deaths annually. At its current trend, AMR is estimated to kill 1 person in every 3 seconds and if no urgent measure is taken, it would cause 10 million death and US$ 100 trillion economic loss every year by 2050 [1]. Antimicrobials are widely used in animals not only for therapeutic or prophylactic purposes but also for non-therapeutic purposes such as growth promotion. The consumption of global antibiotics was increased by 65% between 2000 and 2015, from 21.1 to 34.8 billion defined daily doses (DDDs), while the antibiotic consumption rate increases to 39% from 11.3 to 15.7 DDDs per 1,000 inhabitants per day over the same period [2]. An estimated amount of 131,109 tons of all antimicrobials were used in animal foods in 2013 and the number is projected to rise to 200,235 tons by 2030 [3]. Around 70% antimicrobials intended for human use were sold to use in animal foods in the USA and 30 different countries in Europe. However, there is little to no information on antimicrobial usage in low resource countries [4]. In 2021, World Organization for Animal Health reported an overall decrease of antimicrobial use (34% in the mg of use per kg of production) in the livestock sector at the global level [5]. The antimicrobial quantities dropped from 174.01 mg/kg in 2015 to 114.84 mg/kg in 2017. A projection, based on country-level evaluations of how much data on antimicrobials were captured, also presented the decreasing trend, from 176.71 mg/kg in 2015 to 116.30 mg/kg in 2017 [5]. In 2010, India became the largest consumer of antibiotics (10·7 units per person), associated with the highest burden of infectious diseases [6]. Antifungals should also be carefully used and not rampantly accessible in order to arrest and suppress development of AMR [7]. The aim of the review is to discuss about AMR in poultry, livestock, flying mammals, aquaculture, and human with India's One Health approach to minimize the multi-compartmental gap, the model that can be used in other low-resource settings.

## 2 Root of AMR and classification of resistant pathogens

Antibiotic resistance genes (AGRs) emerged in ancient times in response to naturally occurring antibiotics in the environment, but the severity of progression has been expedited in exposure to the modern usage of antibiotics. Resistance can also be developed spontaneously by mutation. Horizontal gene transfer occurs through inheritance or it can be acquired from non-relatives through mobile genetic elements like plasmids, transposons and integrons [8]. Several pathogenic bacteria related to disease outbreak and few nosocomial bacteria have evolved into multidrug resistant or “superbug” status, such as *Mycobacterium tuberculosis*, *Clostridium difficile, Enterobacter* spp.*, Enterococcus faecium, Enterococcus faecalis, Escherichia coli, Haemophilus influenzae, Klebsiella pneumoniae*, etc. [9]. Some bacteria that are classified as extremely drug resistant (XDR) for their resistance to all or almost all approved antimicrobial agents, like *Streptococcus pneumoniae* and *Enterobacteriaceae* producing extended-spectrum β-lactamases (ESBL). Likewise, pan drug resistance (PDR), the most notorious form of resistance category, is defined as non-susceptibility to all the antimicrobial categories that mankind has discovered to date.

The World Health Organization (WHO) classified the pathogens according to the species and the type of resistance into three priority tiers including critical, high and medium. Some examples of pathogens under the critical tier include *Acinetobacter baumannii, Pseudomonas aeruginosa* and *Enterobacteriaceae* family (*Klebsiella pneumoniae*, *Escherichia coli*, *Enterobacter* spp., *Serratia* spp., *Proteus* spp., *Providencia* spp., and *Morganella* spp.). For pathogens in the high priority tier, examples of pathogens include *Enterococcus faecium*, *Staphylococcus aureus*, *Helicobacter pylori*, *Campylobacter* spp., *Salmonella* spp*.* and *Neisseria gonorrhoeae*. Finally, medium tier bacteria include *Streptococcus* *pneumoniae*, *Haemophilus* *influenzae* and *Shigella* spp.

## 3 Contribution of livestock/food animal sector

It has long been established that the frequency of antibiotic resistance has a direct and proportional relationship with antibiotic consumption [10]. As of late, the WHO has issued a warning that the misuse and overuse of antibiotics in humans and animals is accelerating the AMR progression [11].

Many European countries and the US are the principal users of antibiotics in food animals for therapy or prophylaxis, but they have banned the use of antibiotics as a growth promoter [12]. Government of India also recently banned the manufacture, sale and distribution of colistin and its formulations for food animals, poultry, aquaculture and animal feed supplements [13]. The worldwide use of antimicrobials in livestock consists of 73% of the entire global consumption. Currently, the global consumption of antibiotics is estimated to be 131,000 tons annually which is predicted to increase by more than 67% (approximately 200,000 tons) in 2030 [14]. For Brazil, Russia, India, China, and South Africa (more commonly known as the BRICS countries), the increase of antimicrobial consumption will go up to 99% along with seven times growth in population [15].

It was estimated that the global average annual consumption of antimicrobials per kilogram of animal production will require 45 mg/kg for cattle, 148 mg/kg for poultry and 172 mg/kg for pigs between 2010 and 2030 [15]. The intensive livestock farming has augmented food production at a low cost per unit produced, but possibly an unrecognized price is paid for amplified antimicrobial resistance. India has extremely high prevalence of antimicrobial resistant bacteria in human (e.g., ∼95% of adults in India carry bacteria resistant to β-lactams) and the country is already facing the challenge with antibiotic overuse/misuse both in human and veterinary medicine [16]. Due to the high bacterial infection burden, where antimicrobials play a serious role in reducing mortality and morbidity, the resistance is more crucial in India [17]. A series of scientific studies have reflected how the antibiotic resistance has cropped up in livestock sector in India. Several livestock species, particularly food animals emerged as important reservoir of superbugs such as cattle [[18], [19], [20]], buffalo [21], pig [22,23], poultry [24] and duck [25]. Few antibiotics are usually kept reserved for critical human patients and has not been used in animals in India, such as carbapenem [26] and vancomycin [27]. As of late, pet animals like dogs emerged as reservoirs of carbapenem resistant *Enterobacteriaceae* [28,29]*.* A few clinical conditions like mastitis [19,20], metritis (pyometra) [26] and diarrhea [26] seem to play a role in increased antimicrobial usages and consequently contribute to the generation of resistant bacteria in these conditions. On the other hand, livestock pathogens being resistant to reserved antibiotics indicate the possibility of transfer of resistance genes from the environment.

The activities ultimately pay to the upsurge of contamination in water bodies and environments by wide range of pharmaceutical industries and services as well as discharge from healthcare and livestock settings. Environmental contamination with pharmaceutical wastes encourages transfer of antimicrobial resistance genes among bacterial communities in the environment [30].

Manure is a “hot spot” of bacteria carrying antibiotic resistance genes (ARGs) located in mobile genetic elements (MGE) which can be transferred from one microbe to another by conjugation, transformation or transduction. The capture, accumulation, and dissemination of AGRs are largely dependent on MGEs which permit both intracellular and intercellular DNA mobility. Some examples of identified ARGs of bacteria include β-lactams (*bla*), tetracyclines (*tet*), sulfonamides (*sul*), macrolides (*erm*), aminoglycosides (*aac*), fluoroquinolone (*fca*), colistin (*mcr*), vancomycin (*van*), and multidrug (*mdr*). When soils are treated with manure, antimicrobial agents and their metabolic product as well as bacteria carrying ARGs are introduced into the soil [31]. Moreover, the detection of microorganism carrying emerging ARGs in companion animals, such as dogs and cats, suggests that these animals can also act as reservoirs of resistant bacteria [32]. Household insects, rodents, and pets act as sentinels or bio-indicators for the surveillance of AMR. So, insects, rodents, and pets could be subsequently used as an early warning system for the surveillance of AMR especially in household ahead of its detection in humans [33]. Immediate action should be taken to prevent or minimize the transfer of antimicrobial resistant bacteria from plants and vegetables to animals and finally into human food chain. In this context, collaboration among multiple sectors is essential and this approach is fundamental to implement the concept of One Health.

## 4 Contribution of human health sector

Self-medication/over the counter sale without formal prescription, misuse or overuse and over-prescription of antimicrobials in human clinical medicine are major contributing factors towards the development of AMR [34]. The rate of inappropriate antibiotic use in the primary healthcare setting is as high as 55% in South Africa, 88% in Pakistan, 61% in China, and 15.4% in Canada. China and India represent the largest hotspots of antimicrobial resistance, with new hotspots emerging in Brazil and Kenya. In India, the per capita use of antimicrobials in human increased from 4.40 DDD to 5.74 DDD in the period between 2010 and 2020 [35]. Although the regulatory approach as recommended by the WHO restricted the prescription of antibiotics by qualified physicians, people often receive antibiotics over the counter in some countries without prescription, or with a suggestion from informal providers (i.e. informal prescription), from friends and family members. For example, a study conducted in low-resource settings explored the random selection of antibiotics by informal providers without following the standard treatment guidelines [36].

Other than the overuse and misuse of antibiotics, few critical states of affairs such as invasive surgical procedures, prolonged therapy for chronic infections, immunocompromised patients, and most importantly, the failure of infection prevention and control measures lead to excessive use of antimicrobials resulting in transmission of ARGs from patient to patient and to the environment [37]. There is evidence of a surge in the inappropriate use of antibiotics during the current COVID-19 pandemic [38]. In a multi-hospital cohort study, nearly two-thirds of COVID-19 patients received antibiotics empirically, although only 3.5% had a confirmed bacterial infection [39].

## 5 Contribution of aquatic animal sector

Compared to the antimicrobial use in terrestrial food animal production sector, the application of antimicrobials in aquaculture delivers a potentially wider environmental exposure pathway through water with substantial impact on the ecosystem. By 2030, global antimicrobial use for humans, terrestrial and aquatic food animal sectors are predicted to cumulatively reach 236,757 tons annually. Since 1991, China has contributed to more annual farmed fish output by weight than all other countries combined [40]. In 2017, China (57.9%), India (11.3%), Indonesia (8.6%) and Vietnam (5%) had the largest share of antimicrobial consumption in aquaculture in the Asia-Pacific region. These four countries are expected to continue as the principal consumers of antimicrobials in 2030, with slight or no change in share - China (55.9%), India (unchanged), Indonesia (10.1%) and Vietnam (5.2%) [41]. Indiscriminate antimicrobial use as prophylactic and therapeutic agents in aquaculture can lead to selection, evolution, and horizontal gene transfer of antimicrobial resistant bacteria and antimicrobial resistance genes in the environment [42,43]. The ARGs can be transferred into humans through use of contaminated water for drinking and daily use, and aquatic animal products, suggesting an imperative need for surveillance of AMR.

### 5.1 Wildlife sector

Generally, wild animals do not come into contact with antimicrobials but they may be infected by antimicrobial resistant bacteria from human, agricultural and aquatic sources associated with contaminated environments. Once wild animals acquire resistant bacteria, they can serve as reservoirs/spillovers, vectors and bio-indicators of AMR [44]. Bats act as hosts to a range of viral, bacterial, fungal and parasitic zoonoses. Human activities increase the likelihood of exposure to bats, thereby increasing the opportunity for infections to spill over [45]. The use of antibiotics and other drugs in food animals can directly interfere the health and survival of scavengers and wildlife. Moreover, wild birds are capable of long-range movements and may spread antibiotic resistance across the continents [46,47]. Exploration of AMR in Indian wildlife is limited although the studies conducted in wild crows revealed resistance against quinolones followed by tetracyclines in *Campylobacter* isolates [48]. The captive wild animals kept in different zoological gardens revealed occurrence of multi-drug resistant *Salmonella* in golden pheasant and leopards [49], ESBL-producing *E. coli* in wild birds, deer, zebra, tiger, bear [50], multi-drug resistant, ESBL-producing/carbapenem resistant *E. coli* in rescued sloth bears [51].

### 5.2 Contribution of agriculture sector

Pesticides play an important role in the management of plant/crop diseases as pesticides include biostatic or biocidal chemicals for controlling weeds, rodents, spiders, insects, nematodes, mollusks and microorganisms. Food and Agricultural Organization (FAO) reported that 20%-40% of global crop production is lost due to pests and crop diseases, resulting in over 220 billion USD in losses [52]. Various plants and plant products are degraded by *Aspergillus niger*, *A. flavus*, *A. alliaceus*, *A. carbonarius*, *A. ochraceus* and *A. parasiticus* in different stages like pre-harvest, harvest, processing and handling. Three groups of antibiotics (aminoglycosides, tetracyclines and quinolones) are commonly used in plant production, which are also regularly used therapeutically in animals and humans. The inorganic compounds including copper shared the largest proportion (43%) of total fungicides used, followed by dithiocarbamates (17%) and triazoles/diazoles (11%), however, increasing resistance to fungicides and pesticides is a pressing issue in agricultural sector [[53], [54]].

Fungicide Resistance Action Committee (FRAC), an expert group of Crop Life International provides guidelines for fungicide resistance management and to extend the efficacy of fungicides which are at risk of resistance development in crop agriculture [20].

### 5.3 Concept of resistome

**Fig. 1:**
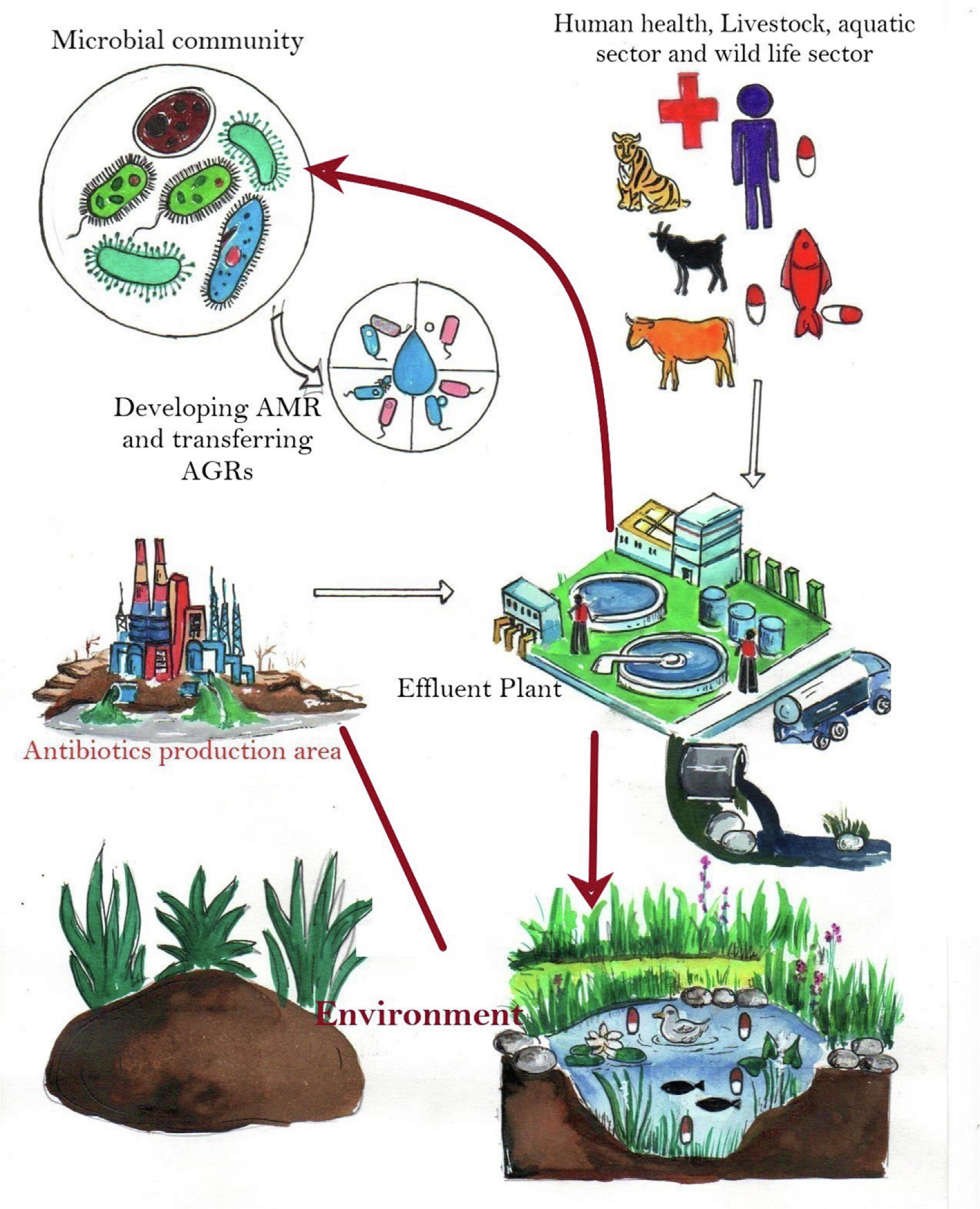
Exchange of antimicrobial resistance genes in different resistome.

The term “antibiotic resistome” was coined by the Wright Lab in 2006 to describe the collection of all antibiotic resistant genes. Soil resistome was described as “resistance determinants present in the soil”. Later, the resistome was defined as “a collection of all the ARGs and their precursors in pathogenic and nonpathogenic bacteria” [55]. The concept of antibiotic resistome (Fig. 1) delivers the knowledge for understanding AMR in the human, animal and environment perspective [56]. India is an exemplar of low-resource settings with high AMR burden. In 2010, India came into the limelight due to the recognition of a new superbug carrying the New Delhi metallo-β-lactamase (NDM-1) gene. It forced to vibrant discussion and more actions on AMR at national and global level [57]. India is the largest consumer of antimicrobials with easy availability of non-prescribed medications over the counter for both human and livestock [58]. High burden of infectious diseases, growing incomes, competition between the human/animal health practitioners, lack of infrastructure related to both human and animal health in remote villages and more dependency on the informal service providers are the key drivers for emergence of AMR [59]. The contamination of water bodies occurred with the antimicrobials discharged from the commercial pharmaceuticals without proper waste water management which was found to be associated with the selection and dissemination of carbapenemase-producing bacteria [60]. China, USA and India are the main producers of non-exclusive active pharmaceutical ingredients (APIs) which makes India the major hub for outsourcing API manufacturing [61].

### 5.4 One Health approach on AMR adapted in a low resource setting

AMR is a global health and development threat which needs urgent multisectoral action in order to avoid the obstacles in achieving the Sustainable Development Goals. Worldwide approaches have been launched by several countries (Table 1.). The approaches taken by India to address AMR can be considered as a model for other low-resource settings. The use of antibiotics for growth promotion in poultry was banned by Bureau of Indian Standards as early as 2007 following the European Union notification [62]. Ministry of Health and Family Welfare, Government of India developed the national policy for containment of antibiotic resistance, which addressed the need of inter-sectoral coordination although the strategies are mostly associated with antibiotic usage in human health sector only [[63], [64]]. The National Livestock Policy promoted judicious use of antibiotics in livestock in India [65]. The withdrawal period of antibiotics in poultry, livestock and sea foods was specified by Drug Controller General of India [66]. Food Safety and Standards Authority of India (FSSAI) also issued the directive regarding the withdrawal period and limit of antibiotic residues in meat and meat products [67]. Finally, based on the Global Action Plan, India's National Action Plan (NAP), a 5-year action plan (2017–2021) for AMR was published in April 2017 by the Union Ministry of Health and Family Welfare as the nodal ministry and the National Centre for Disease Control (NCDC) as the key surveillance body [68]. The livestock, food and environmental sectors were included as strategic priorities in the plan for mitigation of AMR for the first time. The aims of the NAP comprise improving awareness through effective communication, education and training, augmenting surveillance measures, reinforcement infection prevention and control, research and development, encouraging investments, and collaborative activities to control AMR. The major barrier for the implementation of NAP-AMR was detected as lack of national training plan or materials to be used in training program for antibiotic prescribers both in human and animal health sector [69]. On the basis of the NAP, various states have initiated their State Action Plans [70]. Andhra Pradesh, Himachal Pradesh, Kerala, Orissa and Uttar Pradesh were selected as five nodal states to lead the NAP operation process. Kerala was the first state to adopt the sub national State Action Plan in October 2018 followed by Madhya Pradesh, Delhi and Andhra Pradesh.

**Table 1:** Integrated antimicrobial resistance or antimicrobial intake of global and national level surveillance program at a glance.

	Name of the programme	Aims of the programme	Nodal authority
1994	WHONET	To provide free software and training to the laboratories for enabling standardized data collection and analysis	WHO
1995	Danish Integrated Antimicrobial Resistance Monitoring and Research Program (DANMAP)	I. AMR surveillance in human and animal clinical cases, healthy animals, locally produced and imported meat products at wholesale and retail outlets II. Antibiotic consumption surveillance in human and animal hospitals, primary healthcare centers and pharmacies	Ministry of Health and the Ministry of Food, Agriculture and Fisheries, Denmark
1995	Swedish Strategic Programme Against Antibiotic Resistance (STRAMA)	AMR and antibiotic consumption surveillance in human	Sweden
1996	Latin American network for antimicrobial resistance surveillance (ReLAVRA)	Surveillance of resistance in the pathogens isolated from community and hospitals	Pan American Health Organization
1996	Asian network for surveillance of resistant pathogens (ANSORP)	Surveillance of resistance in human hospital associated pathogens (pneumococcus) in Asia	Asia Pacific Foundation for Infectious Diseases
1996	National Antimicrobial Resistance Monitoring System (NARMS)	Monitoring antimicrobial resistance in enteric bacteria from human, retail meat, and food animals	USA
1997	The European Committee on Antimicrobial Susceptibility Testing (EUCAST)	To provide technical aspects of phenotypic *in vitro* antimicrobial susceptibility testing	ECDC, European Society of Clinical Microbiology and Infectious Diseases (ESCMID)
1998	European Antimicrobial Resistance Surveillance Network(EARS-Net)	Data collection from public health laboratories in European Union countries, data management, analysis and validation	European Centre for Disease Prevention and Control (ECDC)
1998	Monitoring of Antimicrobial Resistance and Antimicrobial Use in Animals in Netherlands (MARAN)	AMR and antibiotic consumption surveillance in animals, crops, fruits, vegetables, herbs	Netherlands
1999	Central Asian and Eastern European Surveillance of Antimicrobial Resistance (CAESAR)	Network of national AMR surveillance systems and it comprises all countries of the WHO European Region that are not part of the EARS-Net	WHO
1999	The European Centre for the Study of Animal Health (CEESA)	Surveillance of AMR in healthy livestock at slaughter, clinically sick food and companion animals	ECDC
1999	Japanese Veterinary Antimicrobial Resistance Monitoring System (JVARM)	AMR and antibiotic consumption surveillance in food animals Japan	Japan
1999	National Observatory of the Epidemiology of Bacterial Resistance to Antibiotics (ONERBA)	AMR and antibiotic consumption surveillance in human, food animals, food	France
2000	Global Foodborne Infections Network (GNF)	I. Integrated AMR surveillance. II. Interdivisional coordination among human health, veterinary and food-related disciplines through training courses and events.	WHO
2000	Swedish Veterinary Antimicrobial Resistance Monitoring Programme (SVARM)	AMR and antibiotic consumption surveillance in animals	Sweden
2001	European Surveillance of Antimicrobial Consumption Network (ESAC-Net)	Collection of data on systemic use of antibiotics, antifungals and antivirals in hospital and community in EU countries	ECDC
2001	GermVet	AMR and antibiotic consumption surveillance in food animals	Germany
2003	Italian Veterinary Antimicrobial Resistance Monitoring (ITAVARM)	AMR and antibiotic consumption surveillance in food animals	Italy
2005	CODEX Alimentarius, (CXC 61–2005)	AMR surveillance in humans, food animals, crops and food	FAO & WHO
2005	The Healthcare Associated Infection Network (HAI-Net)	Surveillance of AMR and antimicrobial use in acute and long-term care services	ECDC
2006	Canadian integrated program for antimicrobial resistance surveillance (CIPARS)	AMR and antibiotic consumption surveillance in human, food animals, food items	Canada
2008	Advisory Group on Integrated Surveillance of Antimicrobial Resistance (AGISAR)	I. To establish uniform sampling techniques and methodology for isolation of bacteria and detection of antibiotic resistance II. To afford expert advice III. To stimulate information sharing and supporting capacity-building for AMR surveillance and antimicrobial practice in member country.	WHO
2009	Transatlantic Taskforce on Antimicrobial Resistance (TATFAR)	I. To sponsor appropriate use of antimicrobials in human and veterinary medicine. II. To stop resistant infections in hospital and communities. III. To advance development of new antimicrobials	CDC (USA) and European Union
2010	European Surveillance of Veterinary Antimicrobial Consumption (ESVAC)	Collection of harmonized data on the sales of veterinary antimicrobials (wholesalers, veterinarians, pharmacies)	ECDC
2011	Joint Programming Initiative on Antimicrobial Resistance (JPIAMR)	To assist transnational research and activities in the selected areas such as therapeutics, diagnostics, surveillance, transmission, environment and interventions	European Commission
2012	Viet Nam resistance project (VINARES)	Monitoring antimicrobial resistance in enteric bacteria from human	Viet Nam
2012	Indian network for surveillance of antimicrobial resistance (INSAR)	Monitoring antimicrobial resistance in human	India
2013	Anti-microbial resistance surveillance and research network (AMRSN)	Effective development of a stewardship program	India (ICMR)
2015	Global Antimicrobial Resistance Surveillance System (GLASS)	To give support worldwide surveillance and research in order to reinforce the evidence base on antimicrobial resistance (AMR)	WHO
2017	Indian network for fishery and animals antimicrobial resistance (INFAAR)	Monitoring antimicrobial resistance in livestock, poultry and fishery	ICAR. India
2018	FINRES-Vet	AMR and antibiotic consumption surveillance in food animals	Finland
2020	NethMap	AMR and antibiotic consumption surveillance in human	Netherlands

National Authority for Containment of Antimicrobial Resistance was planned as an extensive body for AMR control activities and a patron in applying the action plan. Indian Council of Agricultural Research (ICAR) has also introduced the Indian Network for Fisheries and Animal Antimicrobial Resistance (INFAAR) with 18 laboratories in different ICAR institutes and State Agriculture/Veterinary Universities (SAUs/SVUs). The network incorporated the advisory committee members from Indian Council of Medical Research (ICMR) and National Centre for Disease Control (NCDC) to look out the problem through the lens of One Health in the true sense. To improve the appropriate use of antibiotics, the NCDC has published the National Treatment Guidelines for Antimicrobial Use in infectious diseases in 2016 and the ICMR published its guidelines in 2017. Food Safety and Standards (contaminants, toxins and residues) Amendment Regulations 2018, has been notified by FSSAI which is related to the tolerance limits of 43 antibiotics in food animal origin products such as meat, milk, poultry, fish, etc. Indian Ministry of Environment, Forest and Climate Change notified an amendment to the Environment (Protection) Rules specific to the Bulk and Formulation on 23^rd^ January, 2020 which specified that maximum residues for as many as 121 antibiotics that can be present in the treated effluent of bulk drug and formulation industry and in the outlet of the Common Effluent Treatment Plant.

India has started strengthening of AMR surveillance network for key pathogens and enrolment in WHO Global Antimicrobial Resistance Surveillance System (GLASS).The WHO-ESBL-*E. coli* Tricycle project on AMR was adapted by India to bridge the research gap in prevalence of AMR in human, animal and environmental sectors with a ‘One Health’ approach in the true sense. Three very recent initiatives have enlightened more the concept of One Health in India: (i) a National Expert Group on One Health as a multi-sectoral trans-disciplinary joint group, (ii) Development of National Institute of One Health at Nagpur, Maharashtra and (iii) Integrated Public Health Laboratories [70]. Central and State governments are gradually taking One Health approaches to confront the fast-emerging issues of antimicrobial resistance, zoonoses and food safety [69]. However, India is still far behind to adapt a mitigation strategy for AMR in wildlife and agriculture sector.

## 6 Conclusion

Numerous One Health initiatives are in progress worldwide while India is nohow behind the ropes. An unfathomable understanding of local issues shall help to figure out the nature of One Health collaborations. The awareness of farmers, livestock managers and environmentalists should be increased regarding the One Health approach and AMR. Incorporation of antimicrobial use, antimicrobial resistance and antibiotic stewardship through One Health approach into medical/veterinary and higher secondary (10 + 2) curriculum may be effective. Indian Medical Association and Indian Veterinary Association can play a significant role to establish a robust One Health approach in India in collaboration with ICMR and ICAR. NCDC can involve a veterinary epidemiologist in the field of epidemiology for One Health epidemiology program. One Health clinics can be adapted at block level for regular close surveillance and reporting system. National animal disease reporting system, Integrated disease surveillance program, Inter-sectoral coordination for prevention and control of zoonotic diseases, National rabies control program and Program for prevention and control of leptospirosis are welcome initiatives, taken by Government of India or NCDC, but more restrictions should be implemented in day-to-day reporting system. A very specialized subject like veterinary public health and community medicine from veterinary and medical sector respectively can give the leadership to establish a strong One Health approach. The domain of One Health covers joint expertise's and action toward AMR, diagnosis of zoonotic diseases, surveillance, epidemiology and their control, food safety, biomedical research, environmental health, production and control of biological products, protection of safe drinking water, and management of public health emergencies like COVID-19 and natural disasters.

## Authors’ contribution

RB and IS conceptualized and prepared the primary draft of the manuscript. CD and SB collected data and critically reviewed the article. All authors read and approved the final manuscript.

## Conflict of interest

The authors declare that there are no conflicts of interest.
